# Synthesis and metal binding properties of *N*-alkylcarboxyspiropyrans

**DOI:** 10.3762/bjoc.13.154

**Published:** 2017-08-04

**Authors:** Alexis Perry, Christina J Kousseff

**Affiliations:** 1 Biosciences, University of Exeter, Stocker Road, Exeter EX4 4QD, UK

**Keywords:** carboxylate ligand, merocyanine, metal binding, photochromism, spiropyran

## Abstract

Spiropyrans bearing an *N*-alkylcarboxylate tether are a common structure in dynamic, photoactive materials and serve as colourimetric/fluorimetric cation receptors. In this study, we describe an efficient synthesis of spiropyrans with 2–12 carbon atom alkylcarboxylate substituents, and a systematic analysis of their interactions with metal cations using ^1^H NMR and UV-visible spectroscopy. All *N*-alkylcarboxyspiropyrans in this study displayed a strong preference for binding divalent metal cations and a modest increase in M^2+^ binding affinity correlated with increased alkycarboxylate tether length.

## Introduction

**Scheme 1:**
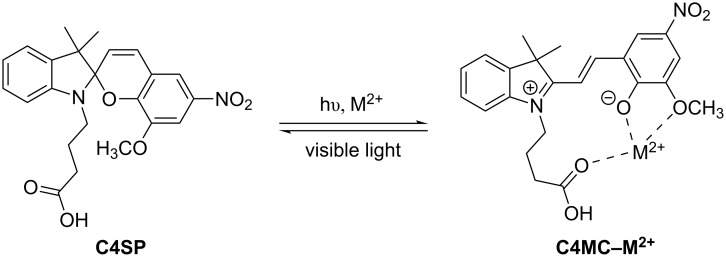
**C4SP–C4MC** spiropyran-merocyanine equilibrium and M^2+^ binding.

Spiropyrans are a class of spiro-fused indolochromene (e.g., **C4SP**, Scheme 1) which exist in photo-controlled equilibrium with their zwitterionic, fully-conjugated merocyanine isomer [1] (e.g., **C4MC**, Scheme 1). The controllable nature of this isomerisation and the dramatic differences in physical and chemical properties displayed by the isomeric forms, coupled with the facile elaboration of the spiropyran core, have made spiropyran–merocyanine systems a common motif in molecular switch and sensing applications [2]. In this respect, the difference in optical properties between colourless, non-fluorescent spiropyran and coloured, fluorescent merocyanine has been extensively exploited [2].

**Figure 1:**
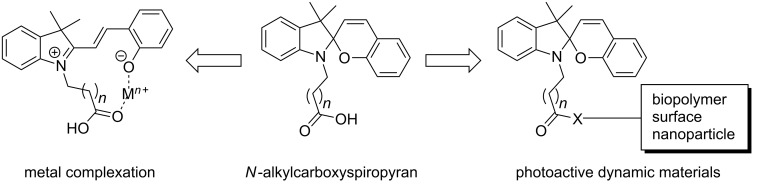
General uses of *N*-alkylcarboxyspiropyrans.

Spiropyrans bearing an *N*-alkylcarboxylate tether are photo-reversible colourimetric/fluorimetric receptors for metal cations [3–5] and amino acids [6], and serve as convenient building blocks in the synthesis of dynamic materials [7] (Figure 1). In this latter role, *N*-alkylcarboxyspiropyrans have been tagged (via ester or amide linkage) to carbon nanotubes (e.g., for photocontrolled Zn^2+^ delivery in biological media [8]) and gold electrodes (for photoswitching the bioelectrocatalytic cascade of cytochrome c/cytochrome oxidase [9]), and attachment to nanoparticles has enabled photomodulation of nanoparticle fluorescence [10–11]. Similarly, tagged *N*-alkylcarboxyspiropyrans have provided the basis for photoactive biopolymers, e.g., polypeptides with photocontrolled folding [12], light-activated enzymes [13], light-enhanced affinity chromatography using spiropyran-modified agarose gel [14–15] and the creation of light-responsive nanopores through modification of natural channel proteins [16]. Furthermore, addition of an *N*-alkylcarboxylate moiety is a common tactic used to enhance water solubility of spiropyran derivatives [6].

Spiropyran–metal cation binding occurs as a result of stabilisation of the zwitterionic merocyanine isomer via phenoxide–metal complexation [17] (Figure 1). Commonly, merocyanines undergo photoreversion to their corresponding spiropyran under visible light irradiation and metal complexation is usually achieved either in darkness or under UV irradiation. Greater stabilisation of merocyanine–metal cation complexes is often achieved through the addition of extra ligation sites and this effect is particularly pronounced in structures bearing an 8′-OMe substituent with respect to binding divalent metal cations [18] (Scheme 1). The extension of this basic bidentate ligand with further substituents has been employed to generate structures with bespoke binding characteristics (e.g., metal ion specificity, control of complex stoichiometry, greater binding affinity) [17]. Typifying this approach, Natali et al. synthesised butanoate-tagged spiropyran **C4SP** and demonstrated its high affinity for Zn^2+^ and Cu^2+^ through the involvement of the *N*-alkylcarboxylate in metal binding [3] (Scheme 1).

Given the evident importance and widespread application of *N*-alkylcarboxyspiropyrans as building blocks in the synthesis of photoactive materials – and as interesting molecules in themselves – we describe herein an efficient synthesis of *N*-alkylcarboxyspiropyrans (analogous to **C4SP**) with alkyl chain lengths from C2 to C12, and a systematic analysis of their metal-binding properties across a range of metal cations. It was envisaged that spiropyrans bearing incrementally-spaced carboxylate groups on conformationally flexible tethers would present trends in their metal-binding characteristics and that such information would have relevance to the application of *N*-alkylcarboxyspiropyrans generally.

## Results and Discussion

### Synthesis

**Scheme 2:**
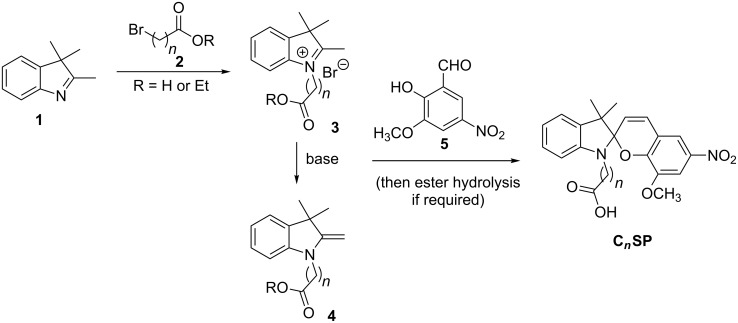
General synthesis of *N*-alkylcarboxyspiropyrans.

Commonly, the synthesis of *N*-alkylcarboxyspiropyrans (e.g., **C4SP**) involves *N*-alkylation of 2,3,3-trimethylindolenine (**1**) with a bromoalkanoic acid or ester **2**, isolation of the resulting indolium salt **3** or treatment with base to generate the *exo*-methylene enamine **4**, then condensation with the appropriate salicylaldehyde **5** (and ester hydrolysis if required) (Scheme 2).

Initially, we followed the conditions employed by Natali et al. in the three-step synthesis of butyric acid derivative **C4SP**, which involved: (i) alkylation of 2,3,3-trimethylindolenine (**1**) with ethyl 4-bromobutyrate (**2**; R = Et, *n* = 3) in chloroform; (ii) condensation of the resulting indolium salt **3** with 3-methoxy-5-nitrosalicylaldehyde (**5**) in ethanol; (iii) basic ester hydrolysis [3] (Scheme 2). Unfortunately, in our hands the alkylation was extremely sluggish, and both this step and the final ester hydrolysis were low yielding. Consequently, we pursued a more efficient approach, reacting trimethylindolenine (**1**) directly with bromoalkanoic acids **2a–g**, obviating the requirement for ester hydrolysis (Table 1). *N*-Alkylation of trimethylindolenine (**1**) with bromoalkanoic acids/esters is often slow and the solvent choice is crucial in identifying viable, robust conditions. Reported protocols have employed chloroform [3], acetonitrile [19], acetone [20], nitromethane [21], 1,2-dichlorobenzene [22], toluene [23], reaction in the absence of solvent [24] or use of microwave irradiation [25]. We found that refluxing acetonitrile was relatively effective, if slow (completion in ca. 60 h), and that a more rapid process occurred if the solvent was allowed to evaporate during the course of the reaction (completion in ca. 20 h). The reaction in the absence of solvent was less effective, perhaps due to inefficient stirring of the small reaction volume (ca. 0.3 mL) or the absence of polar aprotic solvent-mediated acceleration of this S_N_2 process (Table 1, compare entries 6, 7 and 8).

**Table 1:** Synthesis of spiropyrans **C2–C12SP**.

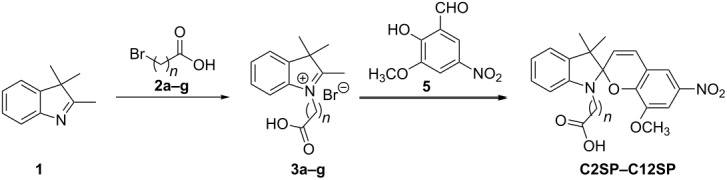						

Entry	*n*	Bromoacid	Alkylation conditions^a^	Condensation conditions^b^	Product	Yield from **1**

1	1	**2a**	method A	–	**C2SP**	0%
2	1	**2a**	method B^c^	method C^c^	**C2SP**	21%
3	2	**2b**	method A	method C	**C3SP**	81%
4	3	**2c**	method A	–	**C4SP**	0%
5	3	**2c**^d^	method A	method D	**C4SP**	38%
6	5	**2d**	method B	method C	**C6SP**	52%
7	5	**2d**	method A	method C	**C6SP**	71%
8	5	**2d**	method A^e^	method C	**C6SP**	48%
9	7	**2e**	method A	method C	**C8SP**	60%
10	9	**2f**	method A	method C	**C10SP**	82%
11	11	**2g**	method A	method C	**C12SP**	67%

^a^Method A: Solution of bromoacid (1 equiv) and 2,3,3-trimethylindolenine (1 equiv) in acetonitrile (0.6 M) heated at 80 °C without a condenser for 20 h; Method B: Solution of bromoacid (1 equiv) and 2,3,3-trimethylindolenine (1 equiv) in acetonitrile (0.6 M) heated at reflux for 72 h. ^b^Method C: Solution of crude indolium salt (1 equiv), salicylaldehyde **5** (1 equiv) and piperidine (1 equiv) in MEK (50 mM) heated at reflux for 20 h; Method D: (i) Solution of crude indolium salt (1 equiv) and salicylaldehyde **5** (1 equiv) in ethanol (0.1 M) heated at reflux for 20 h then (ii) solution of crude spiropyran stirred in 2:1 THF/NaOH (50 mM). ^c^Conducted at room temperature. ^d^Reaction used ethyl 4-bromobutyrate. ^e^Conducted without solvent.

The relative instability of indolium salts is well-documented [26] and our attempts to purify compounds **3** by silica gel chromatography or recrystallisation were unsuccessful. In light of this, and given that our alkylation reactions were relatively clean, crude indolium salts **3** were then condensed with 3-methoxy-5-nitrosalicylaldehyde (**5**) to give the required spiropyrans **C*_n_*SP** (Table 1). Commonly, this has been achieved through simple reflux in ethanol; however, this transformation proved relatively ineffective for alkylcarboxyindolium salts **3** and consequently, we applied alternative conditions using MEK and piperidine [27]. This two-step approach was used to synthesise a range of *N*-alkylcarboxyspiropyrans derived from bromoalkanoic acids of varying chain length in two-step yields from 60–82% (Table 1, entries 3, 7, and 9–11).

**Scheme 3:**
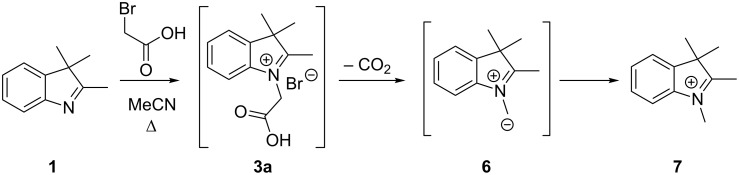
Decarboxylation of *N*-ethanoic acid indolium salt **3a**.

This protocol was ineffective for the synthesis of **C2SP** (*n* = 1) and **C4SP** (*n* = 3). In the former case, the intermediate *N*-ethanoate indolium salt **3a** underwent thermal decarboxylation to give *N*-methylindolium salt **7**, in a similar manner to that previously reported [28]. Presumably, this reaction proceeds via azomethine ylid **6** (Scheme 3); analogous indolium ylids have been used synthetically in 1,3-dipolar cycloadditions [29] and mechanistic studies have been published on the related decarboxylation of pyridinium 2-carboxylates [30]. Fortunately, α-bromocarbonyls such as bromoacetic acid are excellent S_N_2 electrophiles, and *N*-alkylation of 2,3,3-trimethylindolenine (**1**) with bromoacetic acid (**2a**) was successful, if somewhat sluggish, at room temperature. Reaction of the crude indolium salt **3a** with 3-methoxy-5-nitrosalicylaldehyde (**5**) was also conducted at room temperature and generated the ethanoic acid substituted spiropyran **C2SP**, albeit in modest yield (Table 1, entry 2).

**Scheme 4:**
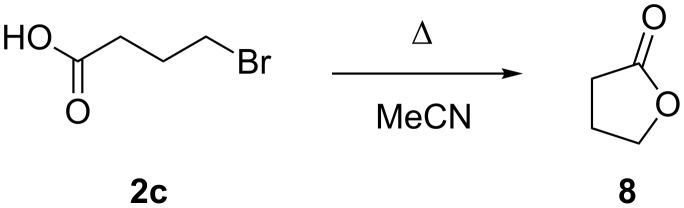
Lactonisation of 4-bromobutyric acid **2c**.

Our attempts to synthesise **C4SP** using our two-step protocol were undermined by the ineffective alkylation of trimethylindolenine (**1**) with 4-bromobutyric acid (**2c**). Under our alkylation conditions, intramolecular lactonisation of 4-bromobutyric acid to γ-butyrolactone (**8**) was more rapid than *N*-alkylation and no indolium product was observed (Scheme 4). Consequently, to obtain **C4SP**, we employed an optimised version of the three-step procedure of Natali et al. [3], wherein lactonisation is avoided through the use of ethyl 4-bromobutyrate, and basic ester hydrolysis is required as a final step (Table 1, entry 5).

### Binding studies

Analysis of spiropyran–merocyanine equilibria with respect to metal binding has frequently employed UV-visible spectroscopy. Merocyanine–metal cation complexes absorb strongly in the visible range (often 450–600 nm), giving rapid access to clear, quantitative data at low concentrations and without interference from paramagnetic transition metal cations (a feature which causes significant problems with NMR analysis). Conversely, use of bound merocyanine–metal cation absorbance intensity as the sole metric for quantification of metal binding of different metal cations should be undertaken with caution. Direct comparison of absorbance intensity measurements (as a surrogate for concentration) is only valid if the molar extinction coefficient, ε, remains constant for all merocyanine complexes of different metals, and indeed for different merocyanine complexes of the same metal. We thus sought initial validation of UV–vis data by direct comparison with data from ^1^H NMR, derived from merocyanine complexes of two non-paramagnetic metal cations, Mg^2+^ and Zn^2+^. ^1^H NMR allows rapid assessment of spiropyran:merocyanine via measurement and comparison of integrals, and hence enables calculation of [merocyanine]. In turn, [merocyanine] can be applied to quantify the corresponding UV data. Related approaches have employed IR spectroscopy [31] and partial least squares regression analysis [32] as adjuncts to UV–vis spectroscopy in the analysis of merocyanine binding across ranges of transition metal cations.

**Figure 2:**
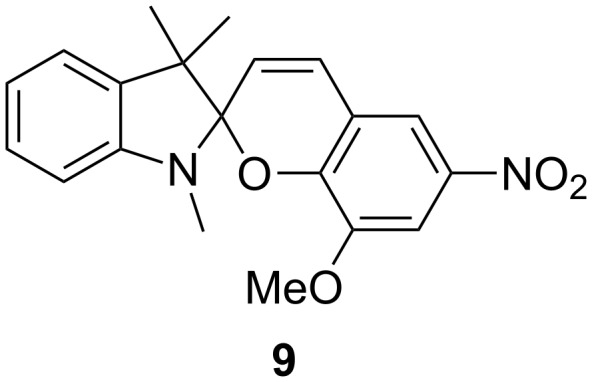
*N*-methyl spiropyran **9**.

We prepared solutions of **C2SP–C12SP** in CD_3_CN to which were added aqueous solutions of either Zn(NO_3_)_2_·6H_2_O or Mg(NO_3_)_2_·6H_2_O. These samples were allowed to equilibrate in darkness at room temperature overnight and were then analysed by ^1^H NMR spectroscopy. Following this, the samples were diluted, allowed to re-equilibrate in darkness, and then analysed by UV–vis spectroscopy. Similar Zn^2+^ and Mg^2+^ samples were prepared with *N*-methyl spiropyran **9** (Figure 2, prepared according to a reported procedure [33]), which served as a carboxylate-free control.

**Figure 3:**
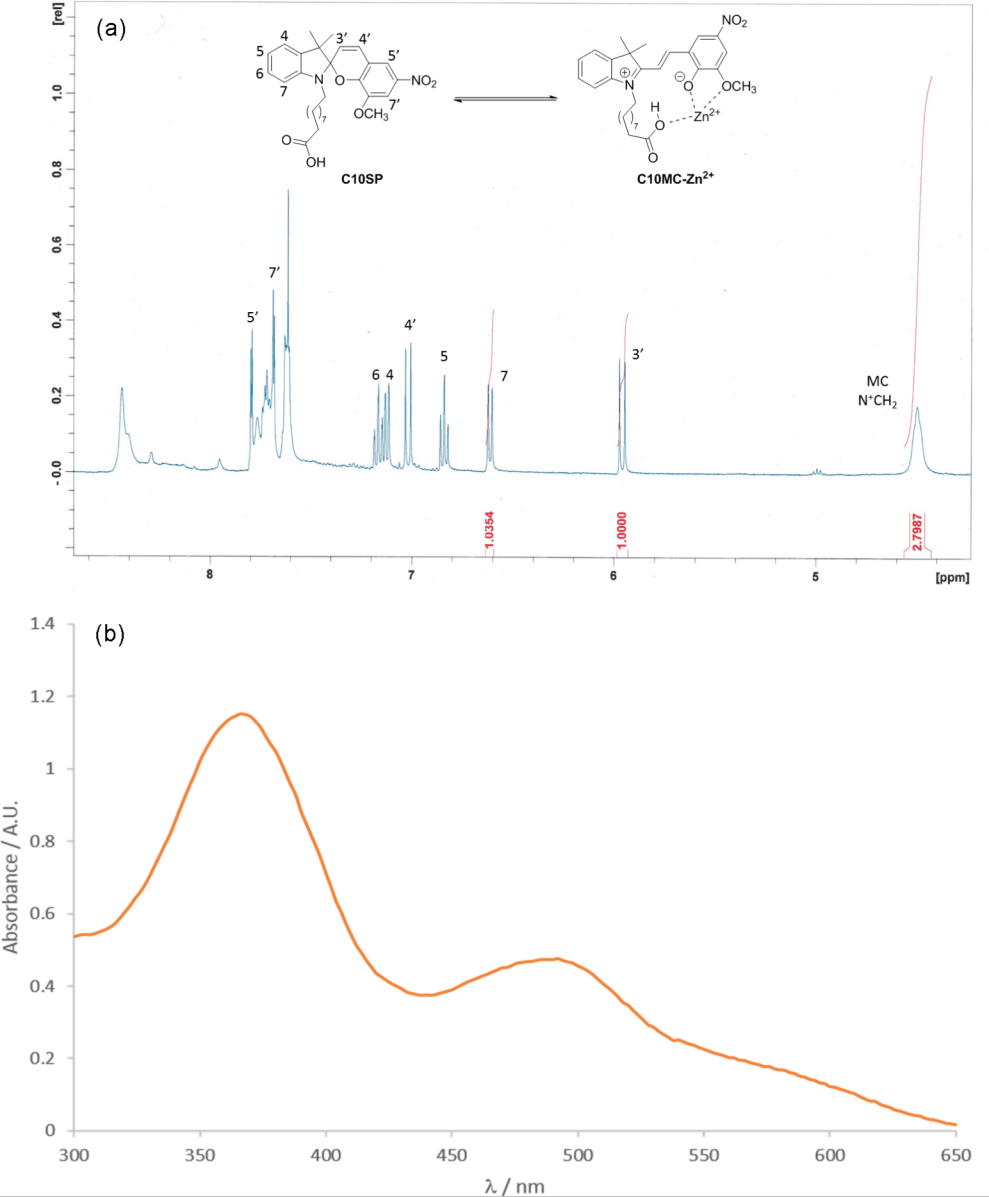
Example spectra illustrating binding studies of spiropyrans with M^2+^. (a) ^1^H NMR spectrum of **C10SP** (10 mM) and Zn(NO_3_)_2_·6H_2_O (2 mM) in CD_3_CN–H_2_O (99.9% v/v) after 18 h in darkness. Peaks corresponding to H nuclei within the spiropyran isomer are labelled; aromatic peaks from the merocyanine–Zn^2+^ complex are broad and unassigned. SP:MC was estimated by comparison of integral values from SP 3′-H and MC (N^+^CH_2_)/2 (as shown in red). (b) UV-visible absorbance spectrum of **C10SP** (0.1 mM) and Zn(NO_3_)_2_·6H_2_O (0.02 mM) in CD_3_CN/CH_3_CN–H_2_O (99.9% v/v) with MC–Zn^2+^ prominent at 495 nm. [**C10MC**–Zn^2+^], derived from (a), was used to calculate of *ε* for this absorbance.

**Figure 4:**
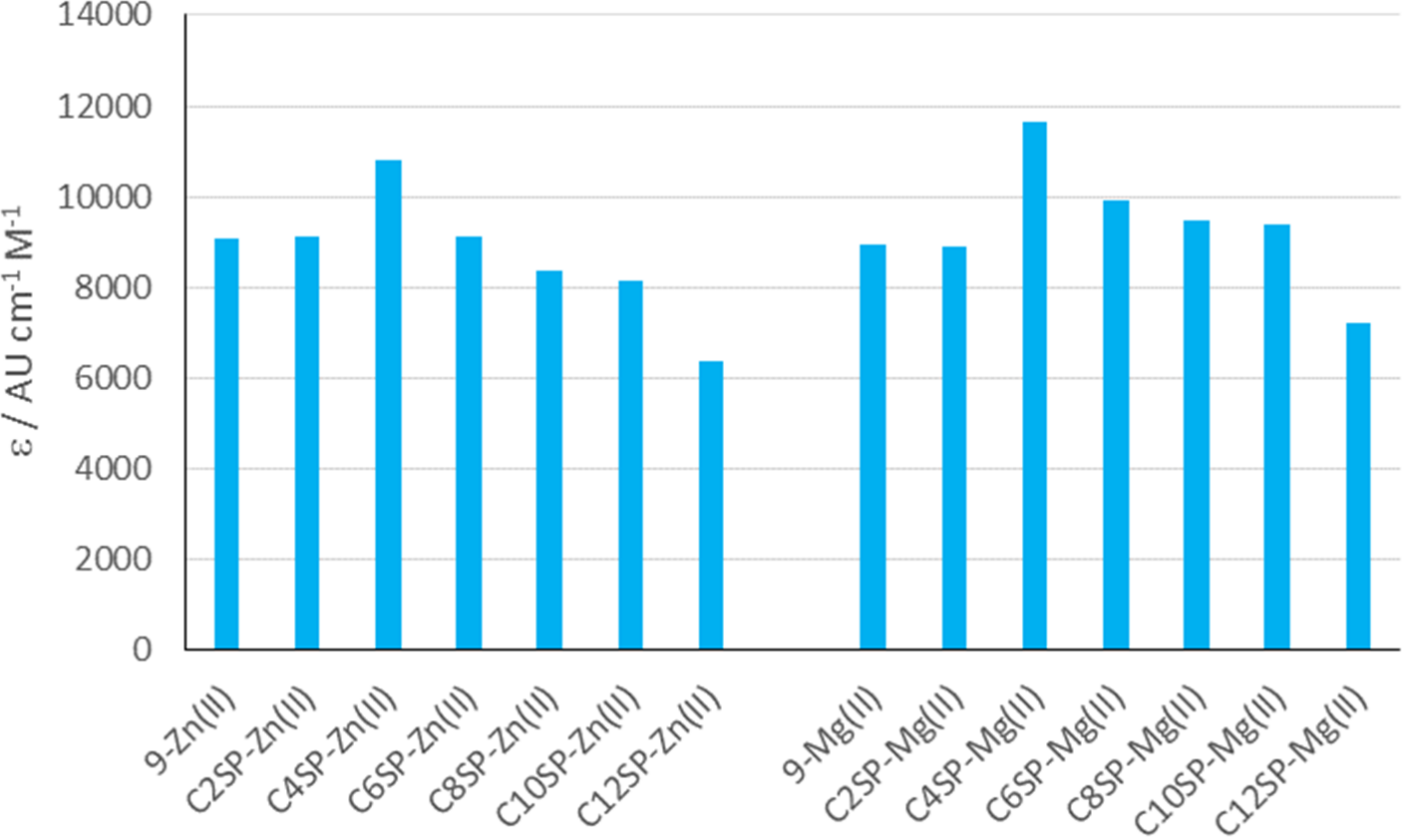
ε for MC–M^2+^ complexes of **C2SP**–**C12SP** and **9**: (left) with Zn^2+^; (right) with Mg^2+^. Values for ε were calculated by application of ^1^H NMR-derived values for [MC–M^2+^] to UV–vis spectroscopy-derived absorbance intensities of MC–M^2+^ (see Figure 3).

The results from these experiments, and example spectra, are shown in Figure 3 and Figure 4, respectively, from which two trends are apparent: (i) ε remains relatively constant for a given compound, irrespective of divalent metal cation; (ii) ε decreases slightly with increasing chain length. Unexpectedly, ^1^H NMR analysis of **C3SP** in the presence of Zn^2+^ and Mg^2+^ produced complex and intractable spectra and it would appear that these metal salts are able to promote degradation of this compound. Irrespective of the precise fate of **C3SP** under these conditions, without NMR evidence of merocyanine formation, this compound was necessarily omitted from this study.

**Figure 5:**
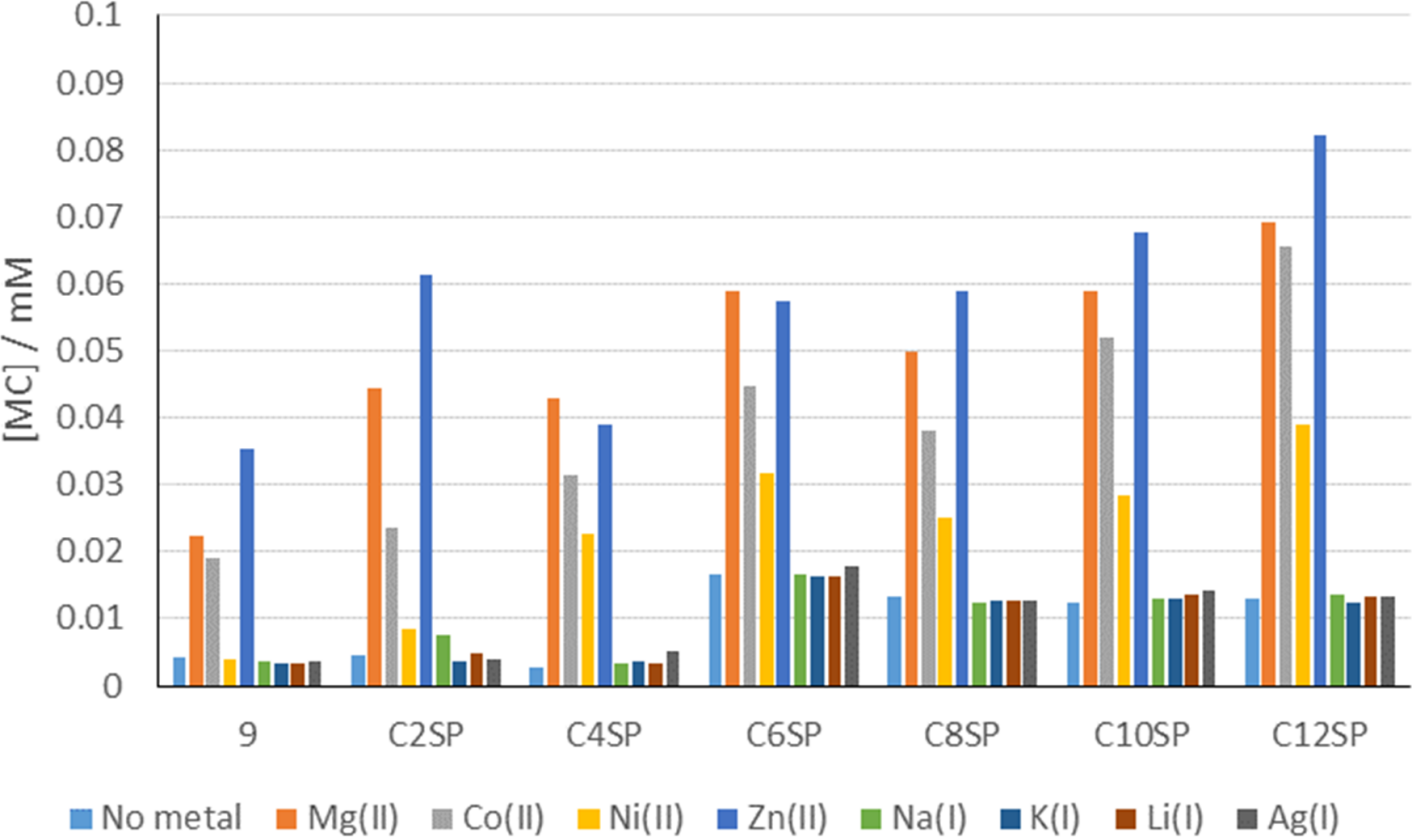
[MC] for compounds **C2SP**–**C12SP** and **9** in the presence of various metal cations. Solutions of spiropyrans (0.1 mM, 1 equiv) and metal nitrates (0.04 mM, 0.4 equiv) in CH_3_CN–H_2_O (99.9% v/v) were kept in darkness for 18 h then analysed by UV–vis spectroscopy. Values for MC absorbance intensity were converted into [MC] using appropriate ε values.

On the basis that the nature of the metal cation appeared to have minimal influence upon the merocyanine extinction coefficient, we were able to use UV–vis spectroscopy to rapidly assess the impact of binding various metal cations upon compounds **C2SP–C12SP** and **9** by applying the NMR-derived ε values (average of ε for MC–Zn^2+^ and MC–Mg^2+^ for each compound) to calculate approximate [merocyanine] from merocyanine absorbance intensity (Figure 5). Although this is based on the analysis of only two metal cations, studies using dynamic modelling have displayed similar, limited impact of different metal cations upon merocyanine ε values [34] so we have cautious confidence in the validity of this approach. All compounds, including control compound **9**, showed selectivity for M^2+^ over M^+^ which follows the pattern previously observed for merocyanines bearing an 8′-OMe substituent [18]. Broadly speaking, the effects upon metal binding of incremental increase in alkylcarboxylate chain length were subtle. The general trends were: (i) compounds bearing longer tethers produced higher concentrations of MC–M^2+^ complex per unit metal ion (presumably on the basis that carboxylates on longer tethers with greater conformational flexibility are able to interact more effectively with cations, despite their inherent entropic penalty [35]); (ii) affinity for metals was in the order Zn^2+^ > Mg^2+^ > Co^2+^ > Ni^2+^. Binding to Ni^2+^ was considerably less effective than for other divalent metal cations and this was particularly pronounced for non-carboxylate **9**, wherein **9**–Ni^2+^ complexation did not exceed background [merocyanine]. Merocyanine complexation of Cu^2+^ was also investigated; however, the absorbance wavelength for MC–Cu^2+^ (≈400 nm) overlapped with that of the larger spiropyran absorbance (≈370 nm) and we were unable to extract meaningful data from this. This is consistent with related studies [3] and, furthermore, spiropyran-based Cu^2+^ detection can be complicated by Cu^2+^-catalysed spiropyran dimerisation [36]. In the light of the results from Natali et al. [3] – in which evidence for involvement of a carboxylate ligand in metal binding was presented but no comparison was made against a control – it is reassuring to note that incorporation of such a ligand into the merocyanine structure did increase the affinity for Zn^2+^, Mg^2+^ and Co^2+^ over that seen in the non-carboxylate control compound **9**.

**Figure 6:**
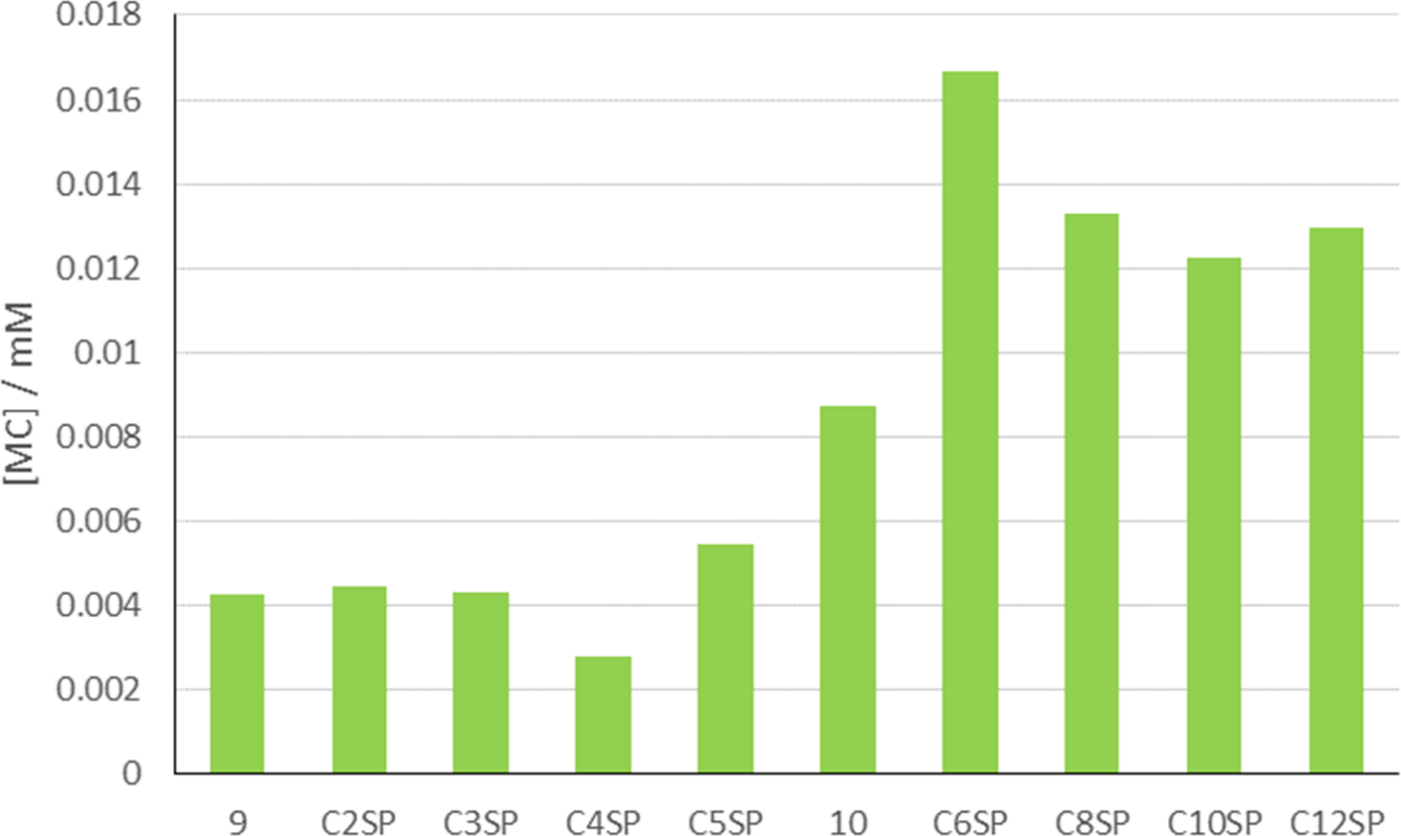
[MC] for spiropyrans **C2SP**–**C12SP**, **9** and **10** (0.1 mM) in CH_3_CN–H_2_O (99.9% v/v). Samples were kept in darkness for 18 h then analysed by UV–vis spectroscopy. Values for MC absorbance intensity were converted into [MC] using appropriate ε values.

**Figure 7:**
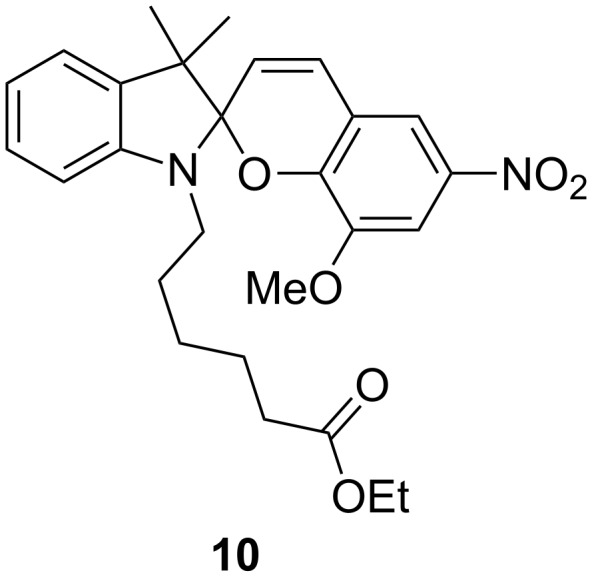
C6 ester derivative **10**.

It is apparent from these data that the length of the carboxylate tether can also affect the spiropyran–merocyanine equilibrium in the absence of metal cations (Figure 5 and Figure 6). All compounds tested existed as mixtures of spiropyran and merocyanine isomers at 0.1 mM in acetonitrile in darkness. For shorter-chain compounds **C2SP** and **C4SP**, and control compound **9**, this resulted in an approximate 2:8 MC:SP ratio. Longer chain carboxylates **C6SP–C12SP**, however, showed higher merocyanine concentrations and these data appear to indicate a sharp threshold between 4-C and 6-C carboxylates. Correspondingly, we synthesised 5-C carboxylate **C5SP** (using our standard protocol; 77%) and assessed its MC:SP equilibrium behaviour under similar conditions. In this case, [**C5MC**] resembled [merocyanine] values for the shorter chain compounds and reinforced the idea of a threshold distance required for carboxylate-mediated merocyanine stabilisation. It is as yet unclear how a long carboxylate tether might stabilise a merocyanine structure whilst structures bearing a superficially similar tether do not. To probe involvement of the carboxylate/carboxylic acid moiety (e.g., in forming inter- or intramolecular hydrogen bonds) we prepared C6 ester derivative **10** (Figure 7) as a direct point of comparison with **C6SP**. In the absence of a hydrogen bond donor group, the concentration of **10MC** at equilibrium is approximately half of that observed for **C6MC** under identical conditions. On the other hand, this value for [**10MC**] is considerably higher than that recorded with shorter chain carboxylates. It would appear, therefore, that tether length is the defining factor in merocyanine stabilisation and that only beyond a threshold distance does hydrogen bond donation of the carboxylic acid become important. This is a key point in designing functional materials based on *N*-alkylcarboxyspiropyrans.

## Conclusion

In summary, we have developed a high-yielding, two-step synthesis of *N*-alkylcarboxyspiropyrans via *N*-alkylation of trimethylindolenine with bromoalkanoic acids, then condensation of the resulting indolium salts with methoxynitrosalicylaldehyde. This protocol is generally effective and enabled the synthesis of a range of spiropyrans bearing different length alkylcarboxylate tethers; however, it is ineffective with bromoacetic acid and bromobutyric acid, where decarboxylation and intramolecular lactonisation respectively compete with *N*-alkylation. In these cases, we have developed alternative, though lower-yielding procedures.

*N*-Alkylcarboxyspiropyrans can function as colourimetric/fluorimetric receptors for metal cations via complexation of the merocyanine isomer. Consequently, we have assessed the metal binding behaviour of spiropyrans bearing *N*-acetic acid through to *N*-dodecanoic acid tethers by ^1^H NMR and UV–vis spectroscopy. All compounds tested displayed a strong preference for divalent over monovalent metal cations and a modest increase in M^2+^ binding affinity correlated with increasing alkycarboxylate tether length.

This paper details a clear, effective protocol for the synthesis of *N*-alkylcarboxyspiropyrans and a thorough analysis of the effect of metal cations upon their spiropyran–merocyanine equilibria. Consequently, the results of this study will impact upon the design and synthesis of dynamic functional materials based on spiropyran–merocyanine units, with particular relevance to cases where such materials are used in the presence of metal cations (e.g., in biological media).

## Supporting Information

File 1 Full experimental details.
