# Supplementary material for: Synthesis and metal binding properties of N-alkylcarboxyspiropyrans
Source: Beilstein J Org Chem. 2017 Aug 4;13:1542–50. doi: 10.3762/bjoc.13.154 (PMC5550821; doi:10.3762/bjoc.13.154)
Supplement: File 1 — Full experimental details. [file Beilstein_J_Org_Chem-13-1542-s001.pdf]

Supporting Information File 1

for

Synthesis and metal-binding properties of *N*-alkylcarboxyspiropyrans

Alexis Perry* and Christina J. Kousseff

Address: Biosciences, University of Exeter, Stocker Road, Exeter EX4 4QD, UK

Email: Alexis Perry* - A.Perry@exeter.ac.uk

* Corresponding author

Full experimental details

Experimental

1. General experimental

UV-visible absorbance spectra were recorded using either a Thermo Scientific Evolution Array spectrophotometer, scanning from 190–1100 nm, or using a Jenway 7315 spectrophotometer, scanning from 200–800 nm. Spectra were recorded in 3 mL quartz cuvettes of 1 cm path length. Infrared spectra were recorded as thin films using attenuated total reflectance with a Nicolet iS5 FTIR spectrometer. Mass spectra were recorded on a QToF 6520 mass spectrometer (Agilent Technologies, Palo Alto, USA). ^{1}H NMR spectra were recorded at either 300 MHz using a Bruker ACF300 spectrometer or at 400 MHz using a Bruker Avance III HD400 spectrometer. ^{13}C NMR spectra were recorded at 75 MHz using a Bruker ACF300 spectrometer. Chemical shifts are quoted in ppm relative to tetramethylsilane, the residual solvent

peak being used for referencing purposes. Coupling constants are quoted to the nearest 0.5 Hz with peak multiplicities for single resonances being labelled as: s, singlet; d, doublet; t, triplet; q, quartet; m, unresolved multiplet. NMR assignments are labelled as follows (using **C3SP** as an example):

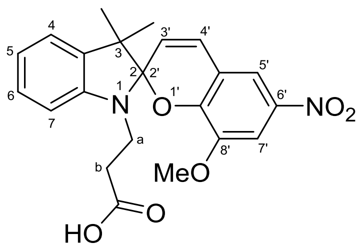

Analytical thin layer chromatography was carried out using Merck Kieselgel 60 F254, coated on aluminium plates, with visualisation of spots where necessary by quenching of UV(254 nm) fluorescence. Silica gel with particle size 40–63 µm was used for flash chromatography.

Solvents and reagents were used as commercially supplied. The fraction of light petroleum ether boiling in the range 40 to 60 °C is referred to as “petrol”. Water refers to deionised water; aqueous solutions were prepared using deionised water.

Spiropyran–merocyanine equilibria are temperature dependent, hence all procedures and measurements were made at 22 °C unless stated otherwise. Incubation of samples in darkness was performed in sealed, foil-wrapped test-tubes or NMR tubes placed in a dark cupboard. *N*-methylated spiropyran **9** was synthesised by a reported procedure [1].

2. Synthesis

Example method for synthesis of *N*-alkylcarboxyspiropyrans:

3-(8′-Methoxy-3,3-dimethyl-6′-nitrospiro[chromene-2′,2-indolin]-1-yl)propanoic acid [2] (C3SP)

A stirred solution of 2,3,3-trimethylindolenine (**1**) (105 µL, 0.653 mmol) and 3-bromopropionic acid (**2b**) (100 mg, 0.653 mmol) in acetonitrile (1 mL) was heated to 80 °C for 20 h, during which time the solvent was allowed to evaporate. The resulting viscous, dark red oil was washed with ethyl acetate (4 × 3 mL) to give crude indolium bromide **3b** which was used directly in spiropyran formation. A stirred solution of **3b**, 3-methoxy-5-nitrosalicyaldehyde (**5**) (129 mg, 0.653 mmol) and piperidine (65 µL, 0.653 mmol) in butanone (10 mL) was heated to reflux for 20 h. The resulting suspension was filtered through a sintered glass funnel, eluting with acetone, and the filtrate was concentrated under reduced pressure. The crude spiropyran was purified by flash chromatography, eluting with 10% petrol in ethyl acetate, ethyl acetate, then 10→50% ethanol in ethyl acetate (the product eluted in several distinct bands). Finally, the product was dissolved in dichloromethane (10 mL) and filtered through paper (to remove silica) to give the spiropyran **C3SP** (218 mg, 81% from **1**) as an amorphous purple solid, spectroscopically identical to that previously described [2].

5-(8′-Methoxy-3,3-dimethyl-6′-nitrospiro[chromene-2′,2-indolin]-1-yl)pentanoic acid (C5SP)

By the same general method, 5-bromovaleric acid (29 mg, 0.159 mmol) gave the *spiropyran* **C5SP** (54 mg, 77%) as an amorphous purple film, R_f 0.2 (ethyl acetate); υ_{max} = 2962, 2851, 1716, 1588, 1517, 1483, 1457, 1334, 1282, 1237 and 1092 cm^{-1}; ^1H NMR (400 MHz, CD_3CN), δ = 7.69 (1H, d, *J*=2.5 Hz, 5′-H), 7.59 (1H, d, *J*=2.5

Hz, 7′-H), 7.07 (1H, t, *J*=7.5 Hz, 6-H), 7.02 (1H, d, *J*=7.5 Hz, 4-H), 6.92 (1H, d, *J*=10.5 Hz, 4′-H), 6.74 (1H, t, *J*=7.5 Hz, 5-H), 6.51 (1H, d, *J*=7.5 Hz, 7-H), 5.87 (1H, d, *J*=10.5 Hz, 3′-H), 3.70 (3H, s, OCH_3), 3.34–3.27 (1H, m, a-C*H*H), 3.20–3.15 (1H, m, a-CH*H*), 2.35–2.20 (2H, m, d-CH_2), 1.72–1.55 (4H, m, b- and c-CH_2), 1.24 (3H, s, 3-CH_3) and 1.20 (3H, s, 3-CH_3); ^{13}C NMR (75 MHz, $CDCl_3$), δ = 178.2 (C=O), 149.4, 147.3, 147.0, 140.3, 136.0, 128.1, 127.6, 122.0, 121.7, 119.2, 118.2, 115.4, 107.9, 106.6, 106.5 (2-C), 56.3 (OCH_3), 52.6 (3-C), 43.2 (a-C), 34.3 (d-C), 28.3, 26.1, 22.3 (3-CH_3) and 19.9 (3-CH_3); HRMS-ES (*m/z*): Found: 439.1884 (MH^+, $C_{24}H_{27}O_6N_2$ requires: 439.1869).

6-(8′-Methoxy-3,3-dimethyl-6′-nitrospiro[chromene-2′,2-indolin]-1-yl)hexanoic acid (**C6SP)**

By the same general method, 6-bromohexanoic acid (**2d**) (246 mg, 1.26 mmol) gave the *spiropyran* **C6SP** (405 mg, 71%) as an amorphous purple film, R_f 0.2 (ethyl acetate); υ_{max} = 2931, 2865, 1717, 1587, 1517, 1484, 1459, 1335, 1283, 1237 and 1092 cm^{-1}; ^{1}H NMR (400 MHz, CD_3CN), δ = 7.79 (1H, d, *J*=2.5 Hz, 5′-H), 7.68 (1H, d, *J*=2.5 Hz, 7′-H), 7.18 (1H, t, *J*=7.5 Hz, 6-H), 7.11 (1H, d, *J*=7.5 Hz, 4-H), 7.02 (1H, d, *J*=10.5 Hz, 4′-H), 6.87 (1H, t, *J*=7.5 Hz, 5-H), 6.63 (1H, d, *J*=7.5 Hz, 7-H), 5.97 (1H, d, *J*=10.5 Hz, 3′-H), 3.79 (3H, s, OCH_3), 3.32–3.22 (1H, m, a-C*H*H), 3.20–3.10 (1H, m, a-CH*H*), 2.35–2.20 (2H, m, e-CH_2), 1.75–1.60 (2H, m, b-CH_2), 1.60–1.48 (2H, m, d-CH_2), 1.50–1.30 (2H, m, c-CH_2), 1.25 (3H, s, 3-CH_3) and 1.18 (3H, s, 3-CH_3); ^{13}C NMR (100 MHz, $CDCl_3$), δ = 178.5 (C=O), 149.4, 147.3, 147.0, 140.3, 136.0, 128.1, 127.6, 122.0, 121.7, 119.1, 118.3, 115.4, 107.9, 106.7, 106.5 (2-C), 56.3 (OCH_3), 52.6 (3-C), 43.4 (a-C), 33.7 (e-C), 28.5, 27.8, 26.7, 24.5 (3-CH_3) and 19.9 (3-CH_3); HRMS-ES (*m/z*): Found: 453.2027 (MH^+, $C_{25}H_{29}O_6N_2$ requires: 453.2025).

8-(8′-Methoxy-3,3-dimethyl-6′-nitrospiro[chromene-2′,2-indolin]-1-yl)octanoic acid (C8SP)

By the same general method, 8-bromooctanoic acid (**2e**) (95 mg, 0.424 mmol) gave the *spiropyran* **C8SP** (175 mg, 60%) as an amorphous purple film, R_f 0.2 (ethyl acetate); υ_{max} = 2929, 2856, 1707, 1587, 1517, 1483, 1458, 1334, 1282, 1236 and 1092 cm^{-1}; ^1H NMR (400 MHz, CD_3CN), δ = 7.79 (1H, d, *J*=2.5 Hz, 5′-H), 7.68 (1H, d, *J*=2.5 Hz, 7′-H), 7.17 (1H, t, *J*=7.5 Hz, 6-H), 7.12 (1H, d, *J*=7.5 Hz, 4-H), 7.02 (1H, d, *J*=10.5 Hz, 4′-H), 6.84 (1H, t, *J*=7.5 Hz, 5-H), 6.61 (1H, d, *J*=7.5 Hz, 7-H), 5.97 (1H, d, *J*=10.5 Hz, 3′-H), 3.79 (3H, s, OCH_3), 3.26 (1H, ddd, *J*=14.5, 7.5 and 7.0 Hz, a-C*H*H), 3.15 (1H, ddd, *J*=14.5, 8.5 and 5.5. Hz, a-CH*H*), 2.25–2.10 (2H, m, g-CH_2), 1.70–1.40 (4H, m, b- and f-CH_2), 1.43–1.22 (6H, m, c-,d- and e-CH_2), 1.25 (3H, s, 3-CH_3) and 1.20 (3H, s, 3-CH_3); ^{13}C NMR (75 MHz, $CDCl_3$), δ = 179.3 (C=O), 149.5, 147.3, 147.1, 140.3, 136.0, 128.0, 127.6, 122.1, 121.6, 119.0, 118.3, 115.4, 107.9, 106.7, 106.5 (2-C), 56.3 (OCH_3), 52.6 (3-C), 43.6 (a-C), 34.0 (g-C), 29.1, 28.8, 28.0, 27.1, 26.1, 24.6 (3-CH_3) and 19.9 (3-CH_3); HRMS-ES (*m/z*): Found: 481.2351 (MH^+, $C_{27}H_{33}O_6N_2$ requires: 481.2338).

10-(8′-Methoxy-3,3-dimethyl-6′-nitrospiro[chromene-2′,2-indolin]-1-yl)decanoic acid (C10SP)

By the same general method, 10-bromodecanoic acid (**2f**) (133 mg, 0.531 mmol) gave the *spiropyran* **C10SP** (261 mg, 82%) as an amorphous purple solid, R_f 0.2 (ethyl acetate); υ_{max} = 2927, 2854, 1707, 1587, 1515, 1482, 1458, 1333, 1282, 1233 and 1092 cm^{-1}; ^1H NMR (300 MHz, $CDCl_3$), δ = 7.71 (1H, d, *J*=2.5 Hz, 5′-H), 7.62 (1H, d, *J*=2.5 Hz, 7′-H), 7.18 (1H, t, *J*=7.5 Hz, 6-H), 7.08 (1H, d, *J*=7.5 Hz, 4-H), 6.87 (1H,

d, *J*=10.5 Hz, 4′-H), 6.85 (1H, t, *J*=7.5 Hz, 5-H), 6.57 (1H, d, *J*=7.5 Hz, 7-H), 5.84 (1H, d, *J*=10.5 Hz, 3′-H), 3.75 (3H, s, OCH_3), 3.27–3.08 (2H, m, a-CH_2), 2.32 (2H, t, *J*=7.0 Hz, i-CH_2), 1.73–1.42 (4H, m, b- and h-CH_2), 1.40–1.20 (10H, m, c–g-CH_2), 1.25 (3H, s, 3-CH_3) and 1.20 (3H, s, 3-CH_3); ^{13}C NMR (75 MHz, $CDCl_3$), δ = 179.3 (C=O), 149.6, 147.4, 147.1, 140.2, 136.0, 128.0, 127.5, 122.2, 121.6, 118.9, 118.3, 115.4, 107.9, 106.7, 106.5 (2-C), 56.3 (OCH_3), 52.6 (3-C), 43.6 (a-C), 34.1 (i-C), 29.6, 29.5, 29.0, 28.9, 28.2, 27.2, 27.0, 25.1 (3-CH_3) and 19.9 (3-CH_3); HRMS-ES (*m/z*): Found: 509.2656 (MH^+, $C_{29}H_{37}O_6N_2$ requires: 509.2654).

12-(8′-Methoxy-3,3-dimethyl-6′-nitrospiro[chromene-2′,2-indolin]-1-yl)dodecanoic acid (C12SP)

By the same general method, 12-bromododecanoic acid (**2g**) (161 mg, 0.575 mmol) gave the *spiropyran* **C12SP** (255 mg, 67%) as an amorphous purple solid, R_f 0.2 (ethyl acetate); υ_{max} = 2924, 2852, 1707, 1586, 1516, 1484, 1457, 1333, 1282, 1234 and 1092 cm^{-1}; ^{1}H NMR (400 MHz, CD_3CN), δ = 7.79 (1H, d, *J*=2.5 Hz, 5′-H), 7.70 (1H, d, *J*=2.5 Hz, 7′-H), 7.20 (1H, t, *J*=7.5 Hz, 6-H), 7.18 (1H, d, *J*=7.5 Hz, 4-H), 7.03 (1H, d, *J*=10.5 Hz, 4′-H), 6.85 (1H, t, *J*=7.5 Hz, 5-H), 6.62 (1H, d, *J*=7.5 Hz, 7-H), 5.97 (1H, d, *J*=10.5 Hz, 3′-H), 3.80 (3H, s, OCH_3), 3.35–3.20 (1H, m, a-C*H*H), 3.18–3.08 (1H, m, a-CH*H*), 2.38–2.20 (2H, m, k-CH_2), 1.70–1.45 (4H, m, b- and j-CH_2), 1.45–1.23 (14H, m, c–i-CH_2), 1.25 (3H, s, 3-CH_3) and 1.19 (3H, s, 3-CH_3); ^{13}C NMR (75 MHz, $CDCl_3$), δ = 179.4 (C=O), 149.6, 147.4, 147.1, 140.2, 136.0, 128.0, 127.5, 122.2, 121.6, 118.9, 118.3, 115.4, 107.9, 106.7, 106.5 (2-C), 56.3 (OCH_3), 52.6 (3-C), 43.6 (a-C), 34.3 (k-C), 29.5, 29.3, 29.0, 28.9, 28.7, 28.1, 27.3, 26.2, 24.8 (3-CH_3) and 19.9 (3-CH_3); HRMS-ES (*m/z*): Found: 537.2970 (MH^+, $C_{31}H_{41}O_6N_2$ requires: 537.2964).

2-(8′-Methoxy-3,3-dimethyl-6′-nitrospiro[chromene-2′,2-indolin]-1-yl)acetic acid (C2SP)

A solution of 2,3,3-trimethylindolenine (**1**) (63 µL, 0.393 mmol) and 2-bromoacetic acid (**2a**) (55 mg, 0.393 mmol) in acetonitrile (1 mL) was stirred at room temperature for 72 h then concentrated under reduced pressure without heating. The resulting viscous, dark red oil was washed with ethyl acetate (4 × 3 mL) to give crude indolium bromide **3a** which was used directly in spiropyran formation. A solution of **3a**, 3-methoxy-5-nitrosalicyaldehyde (**5**) (77 mg, 0.393 mmol) and piperidine (39 µL, 0.393 mmol) in butanone (6 mL) was stirred for 40 h then concentrated under reduced pressure. The resulting crude spiropyran was purified by flash chromatography, eluting with dichloromethane, 10% methanol in dichloromethane then 20% methanol in dichloromethane (the product eluted in several distinct bands). Finally, the product was dissolved in dichloromethane (5 mL) and filtered through paper (to remove silica) to give the *spiropyran* **C2SP** (44 mg, 21% from **1**) as an amorphous red-purple solid, R_f 0.2 (ethyl acetate); υ_{max} = 2963, 2862, 1717, 1607, 1583, 1517, 1484, 1457, 1333, 1282 and 1092 cm^{-1}; ^1H NMR (300 MHz, $CDCl_3$), δ = 7.61 (1H, d, *J*=2.5 Hz, 5′-H), 7.52 (1H, d, *J*=2.5 Hz, 7′-H), 7.11 (1H, t, *J*=7.5 Hz, 6-H), 7.02 (1H, d, *J*=7.5 Hz, 4-H), 6.84 (1H, t, *J*=7.5 Hz, 5-H), 6.81 (1H, d, *J*=10.5 Hz, 4′-H), 6.42 (1H, d, *J*=7.5 Hz, 7-H), 5.84 (1H, d, *J*=10.5 Hz, 3′-H), 4.02 (1H, d, *J*=18.0 Hz, NC*H*H), 3.87 (1H, d, *J*=18.0 Hz, NCH*H*), 3.63 (3H, s, OCH_3), 1.22 (3H, s, 3-CH_3) and 1.13 (3H, s, 3-CH_3); ^{13}C NMR (75 MHz, $CDCl_3$), δ = 174.2 (C=O), 148.4, 147.4, 145.5, 140.9, 135.5, 128.9, 127.8, 121.9, 121.5, 120.4, 118.2, 115.2, 107.8, 106.6, 106.0 (2-C), 56.1 (OCH_3), 52.9 (3-C), 45.2 (NCH_2), 26.2 (3-CH_3) and 19.9 (3-CH_3); HRMS-ES (*m/z*): Found: 397.1402 (MH^+, $C_{21}H_{21}O_6N_2$ requires: 397.1390).

4-(8′-Methoxy-3,3-dimethyl-6′-nitrospiro[chromene-2′,2-indolin]-1-yl)butanoic acid [3] (C4SP)

A stirred solution of 2,3,3-trimethylindolenine (**1**) (202 µL, 1.26 mmol) and ethyl 4-bromobutyrate (180 µL, 1.26 mmol) in acetonitrile (2 mL) was heated to 80 °C for 20 h, during which time the solvent was allowed to evaporate. The resulting viscous, dark red oil was washed with ethyl acetate (4 × 5 mL) to give crude indolium bromide **3c** which was used directly in spiropyran formation. A stirred solution of **3c** and 3-methoxy-5-nitrosalicyaldehyde (**5**) (248 mg, 1.26 mmol) in ethanol (10 mL) was heated to reflux for 20 h then concentrated under reduced pressure. The resulting residue was dissolved in THF (12 mL) to which was added NaOH (6 mL of a 10% aqueous solution) and stirred for 18 h. The reaction mixture was acidified with glacial acetic acid (2 mL) and extracted with dichloromethane (3 × 10 mL). The combined organic fractions were washed with $NaHSO_3$ (10 mL of a 15% aqueous solution), dried ($MgSO_4$) and concentrated under reduced pressure. The resulting crude product was purified by flash chromatography, eluting with 10% petrol in ethyl acetate, ethyl acetate, then 10→50% methanol in ethyl acetate (the product eluted in several distinct bands). Finally, the product was dissolved in dichloromethane (10 mL) and filtered through paper (to remove silica) to give the spiropyran **C4SP** (203 mg, 38% from **1**) as an amorphous purple solid, spectroscopically identical to that previously described [3].

Ethyl 6-(8′-methoxy-3,3-dimethyl-6′-nitrospiro[chromene-2′,2-indolin]-1-yl)hexanoate (10)

A stirred solution of 2,3,3-trimethylindolenine (**1**) (202 µL, 1.26 mmol) and ethyl 6-bromohexanoate (224 µL, 1.26 mmol) in acetonitrile (2 mL) was heated to 80 °C for

20 h, during which time the solvent was allowed to evaporate. The resulting viscous, dark red oil was washed with ethyl acetate (4 × 5 mL) to give the corresponding crude indolium bromide which was used directly in spiropyran formation. A stirred solution of this bromide and 3-methoxy-5-nitrosalicyaldehyde (**5**) (248 mg, 1.26 mmol) in ethanol (10 mL) was heated to reflux for 20 h then concentrated under reduced pressure. The resulting crude product was purified by flash chromatography, eluting with 10% ethyl acetate in petrol, 50% ethyl acetate in petrol, ethyl acetate, then 10% ethanol in ethyl acetate (the product eluted in several distinct bands). Finally, the product was dissolved in dichloromethane (10 mL) and filtered through paper (to remove silica) to give the *spiropyran* **10** (260 mg, 43% from **1**) as an amorphous purple solid, R_f 0.2 (ethyl acetate); υ_{max} = 2933, 2866, 1728, 1655, 1605, 1517, 1481, 1456, 1333, 1283 and 1090 cm^{-1}; ^{1}H NMR (400 MHz, CD_3CN), δ = 7.79 (1H, d, *J*=2.5 Hz, 5′-H), 7.68 (1H, d, *J*=2.5 Hz, 7′-H), 7.12 (1H, td, *J*=7.5 and 1.0 Hz, 6-H), 7.10 (1H, dd, *J*=7.5 and 1.0 Hz, 4-H), 7.03 (1H, d, *J*=10.5 Hz, 4′-H), 6.86 (1H, t, *J*=7.5 Hz, 5-H), 6.63 (1H, d, *J*=7.5 Hz, 7-H), 5.97 (1H, d, *J*=10.5 Hz, 3′-H), 4.09 (2H, q, *J*=7.0 Hz, OCH_2), 3.79 (3H, s, OCH_3), 3.26 (1H, ddd, *J*=15.0, 8.5 and 7.0 Hz, a-C*H*H), 3.18 (1H, ddd, *J*=15.0, 8.5 and 5.5 Hz, a-CH*H*), 2.25–2.10 (2H, m, e-CH_2), 1.72–1.60 (2H, m, b-CH_2), 1.60–1.50 (2H, m, d-CH_2), 1.45–1.30 (2H, m, c-CH_2), 1.25 (3H, s, 3-CH_3), 1.22 (3H, t, *J*=7.0 Hz, CH_2CH_3) and 1.19 (3H, s, 3-CH_3); ^{13}C NMR (75 MHz, $CDCl_3$), δ = 173.6 (C=O), 149.5, 147.4, 147.0, 140.3, 136.0, 128.1, 127.6, 122.1, 121.7, 119.1, 118.3, 115.4, 107.8, 106.6, 106.5 (2-C), 60.2 (OCH_2), 56.2 (OCH_3), 52.6 (3-C), 43.4 (a-C), 34.2 (e-C), 28.6, 26.8, 26.1, 24.7 (3-CH_3), 19.9 (3-CH_3) and 14.2 (CH_2CH_3); HRMS-ES (*m/z*): Found: 481.2339 (MH^+, $C_{27}H_{33}O_6N_2$ requires: 481.2338).

3. Determination of extinction coefficients using ^{1}H NMR and UV-visible spectroscopy

Solutions of either $Zn(NO_3)_2{\cdot}6H_2O$ or $Mg(NO_3)_2{\cdot}6H_2O$ (0.6 µL of 2 M aqueous, 1.2 µmol, 0.2 equiv) were added to solutions of spiropyrans (0.6 mL of 10 mM in acetonitrile-d_3, 6 µmol, 1 equiv). The resulting solutions were shaken, kept in darkness for 18 h then analysed by ^{1}H NMR spectroscopy. For analysis by UV-visible spectroscopy, the samples required dilution to 0.1 mM (with respect to initial [SP]): 0.1 mL of the NMR sample was diluted to 10 mL with acetonitrile, 9.9 µL of water was added, the samples were shaken and kept in darkness for 18 h then analysed by UV-visible spectroscopy.

4. Analysis of metal binding using UV-visible spectroscopy

Solutions of $Zn(NO_3)_2{\cdot}6H_2O$, $Mg(NO_3)_2{\cdot}6H_2O$, $Ni(NO_3)_2{\cdot}6H_2O$, $Co(NO_3)_2{\cdot}6H_2O$, $NaNO_3$, KNO_3, $LiNO_3$ or $AgNO_3$ (2 µL of 0.04 M aqueous, 0.08 µmol, 0.4 equiv), or water (2 µL), were added to solutions of spiropyrans (2 mL of 0.1 mM in acetonitrile, 0.2 µmol, 1 equiv). The resulting solutions were shaken and kept in darkness for 18 h, then analysed by UV-visible spectroscopy.

5. References for Supporting Information

1. Roxburgh, C. J.; Sammes, G.; Abdullah, A. *Dyes Pigments* **2011,** *90,* 146–162.

2. Karube, I.; Yamazaki, M.; Matsuoka, H.; Suzuki, S. *Chem. Lett.* **1983,** *12,* 691–692.

3. Natali, M.; Aakeroy, C.; Desper, J.; Giordani, S. *Dalton Trans.* **2010,** *39,* 8269–8277.
